# News

**DOI:** 10.1155/S1463924601000037

**Published:** 2001


### News

### Chem eStandards™ initiative completes its mission: CIDX™ poised to support ongoing efforts

*New York, 29 January 2001*: Beth Hough, interim executive director of the Chemical Industry Data Exchange (CIDX™), announced today that the second phase of the Chem eStandards™ initiative is complete. Begun in July 2000, the initiative was jump-started by BASF, The Dow Chemical Company and DuPont to develop a broad set of non-proprietary, eXtensible Markup Language (XML) standards to facilitate business-to-business data exchange across the chemical industry worldwide.

The initiative's second phase focused on logistics, invoicing, forecasting, exchange and multinational interactions. As a result of efforts during Phases 1 and 2, over 700 data elements have been defined and 47 new transactions can be conducted using the standards. More than 1000 pages of standards documentation containing technical and implementation information have been created.

Now ready for use, the standards are the result of active contribution by numerous chemical companies and their partners. Additional companies contributing to the project include chemical industry leaders—Air Products, BP, Celanese, Eastman Chemical Company, Occidental Chemical Corporation, Rohm and Haas, Shell and Solutia; chemical marketplaces—CheMatch, ChemConnect, ElastomerSolutions, Elemica and Envera; and service and solution providers—Accenture (formerly known as Andersen Consulting), Bulknet, JP Morgan Treasury Services, Citigroup, Crossworlds Software, Inc., EC Outlook, Extricity, Inc., OneChem Ltd., Transentric, webMethods, Inc., and XMLSolutions Corporation. As of 22 January 2001, other companies that have registered their support and encouragement of the use of these standards are: Arch Chemicals, Inc., Ashland Distribution Company, Atofina, Bayer Corporation, ChemUnity, Clariant International Ltd., ExxonMobil Chemical, The Lubrizol Corporation, NOVA Chemicals and Rhodia Inc.

> 'The collaborative effort of so many industry leaders made the project a resounding success,' said Hough. 'Suppliers and their customers can use these standards as a framework, to reduce implementation times and the costs associated with business-to-business integration.'

Vendor-neutral and available free of charge to all chemical industry members, the standards will continue to be enhanced in the future.

According to Ken Hutcheson, standards director for CIDX™, 'While the Chem eStandards™ initiative may have achieved its initial goal, building support for the standards and developing them further is an ongoing process. These responsibilities are being assumed by CIDX™, where we will have the opportunity to pull together ideas and experiences from across the entire industry.'

Recently, CIDX™ expanded its mission to become the chemical industry's standards body. To fulfil its new role, the group has restructured its organization to include three full-time, on-loan positions and volunteer working committees.

From 5–8 February CIDX™ will hold a meeting in Clearwater, Florida, where attendees will discuss Chem eStandards™ support. Educational sessions, in addition to the initial convening on work committees, are also planned. For information on the meeting or to participate in the proceedings, visit the CIDX™ Web site at http://www.cidx.org. Current versions of the Chem eStandards™ also can be found at this Web site.

The Chem eStandards™ initiative is a multicompany-funded undertaking dedicated to the development and promotion of non-proprietary, XML-based standards for conducting global e-business in the chemical industry. Participating and contributing to this initiative are leading chemical companies, chemical marketplaces and services providers.

The Chem eStandards™ effort is open to all interested parties. Companies interested in participating in the development of Chem eStandards™ should send an e-mail to Info@ChemeStandards.com. Chem eStandards™ are being developed in compliance with applicable laws, including antitrust laws, and pursuant to a standard intellectual property rights policy ensuring open access and use, while at the same time preserving contributing companies' individual rights to their own intellectual property.

*For further information please contact: Shahla Siddiqi, Deardorff Associates. Tel: + 1 302 764-7573, extn 30; e-mail: ssiddiqi@deardorffassociates.com*

DOI: 10.1080/14639240110042995

